# The endothelial specific isoform of type XVIII collagen correlates to annual bleeding rate in haemophilia patients

**DOI:** 10.1371/journal.pone.0190375

**Published:** 2018-01-10

**Authors:** Nadja Gad Kjeld, Baolai Hua, Morten Asser Karsdal, Shu Sun, Tina Manon-Jensen

**Affiliations:** 1 Nordic Bioscience A/S, Herlev Hovedgade, Herlev, Denmark; 2 Department of Hematology, Peking Union Medical College Hospital (PUMCH), Chinese Academy of Medical Sciences (CAMS) & Peking Union Medical College (PUMC), Beijing, China; 3 Department of Hematology, Northern Jiangsu People's Hospital, Yangzhou, Jiangsu, China; University of Insubria, ITALY

## Abstract

**Introduction:**

The medical need in the haemophilic (HF) field to reduce bleeding incidents requires measurement of the annual bleeding rate (ABR) in haemophiliacs. Vascular rupture is associated with damage to the vascular endothelium causing exposure of the basement membrane. Endothelial cells and matrix impairment may be associated with joint bleeds and later development of HF arthropathy. Imbalanced extracellular matrix turnover is a central pathological feature in many diseases consequent to epithelial or endothelial cell damage. Type XVIII collagen is an essential basement membrane component, with an endothelial specific isoform.

**Aim:**

To quantify the basement membrane specifically for the endothelial cells, as that may have particular relevance to endothelial cell stability and rupture in haemophiliacs. A newly developed ELISA assay detecting endothelial type XVIII collagen (COL-18N) was used to assess the clinical relevance of endothelial basement membrane turnover in patients diagnosed with HF arthropathy and correlation to ABR.

**Methods:**

We developed an ELISA assay for quantification of COL-18N. Serum from 35 male HF patients was investigated using the COL-18N ELISA.

**Results:**

COL-18N correlated to the ABR of haemophiliacs, r = 0.45, P<0.006.

**Conclusion:**

Vascular rupture and consequent bleeding are associated with joint damage and deterioration of life quality in haemophiliacs. Quantification of ABR is an important part in efficacy assessment of different interventions, and the benchmark of these. Objective biomarkers reflecting endothelial dysfunction, vascular leaks and rupture, like the COL-18N biomarker that associate with ABR, may assist in identifying the most optimal treatment and monitoring of HF patients.

## Introduction

Recurrent haemarthroses consequent to vascular ruptures is a major complication in haemophilia, contributing to progressive joint damage, which leads to haemophilic (HF) arthropathy. Although a crude measure, the annual bleeding rate (ABR) is associated with HF arthropathy [1] but is also a key parameter in clinical trials ensuring quantifiable benefits to patients [2–4]. Endothelial cells impairment and matrix quality may be associated with joint bleeds and later the development of HF arthropathy. While the endothelial cell function is debated, no quantifiable methods are available for specifically quantifying damage to the vascular endothelium, which subsequent to bleeding, results in exposure of the basement membrane underlying the endothelial cells.

**Fig 1:**
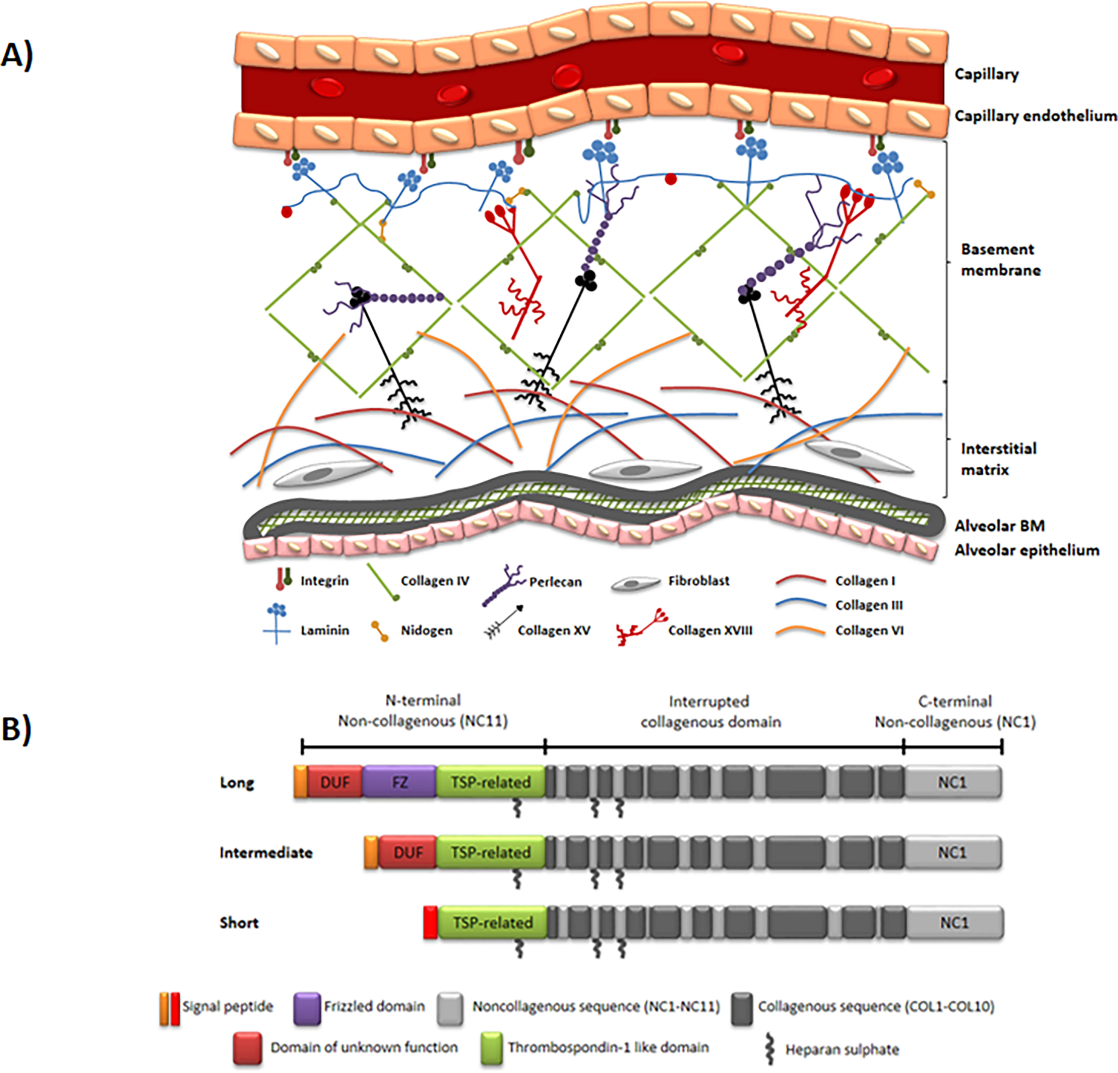
Type XVIII collagen location and structure. (A) Structure of the vascular basement membrane (BM). The capillary subendothelial layer is composed of a BM and an interstitial matrix (IM). The main components of vascular basement membranes include type IV collagen, laminin and nidogen. Minor components include type XV collagen and type XVIII collagen. The components of the BM self-assemble into sheet-like structures. The BM is tightly connected to the IM through interactions between collagen type I and VI and collagen type IV and XV. (B) Isoforms of Type XVIII collagen. Collagen type XVIII exists in three isoforms, which differ in their N-terminus. The *COL18A1* gene encodes these variants by the use of two promoters and alternative splicing. The short isoform has a different signal peptide from the other two, and coded by promoter 1, while the others have the same signal peptide and both coded by promoter 2. All isoforms include a thrombospondin-like domain, heparin sulphate chains and a globular C-terminal containing the type XVIII collagen fragment, endostatin.

Collagen IV, XV and XVIII represent the most well-known collagens of the vascular basement membrane, responsible for maintaining vessel wall structure and integrity of the membrane (Fig 1A) [5–7]. Type XVIII collagen exists in three isoforms: short, intermediate, and long, localized in various basement membrane zones [8–10] (Fig 1B). The short isoform is found in blood vessels and around muscular structures. Here, zero or only very low amounts of the intermediate and long isoforms are present [9].

The role of type XVIII collagen is highlighted by the clinical consequences of mutations in this protein, leading to the autosomal recessive disorder Knobloch syndrome (KS). KS is characterized by various eye defects leading to blindness at a young age [11,12]. Moreover, *col18a1-/-* knock-out mice showed delayed regression of blood vessels in the vitreous along the surface of the retina, impaired angiogenesis of retinal vessels and altered iris basenment membrane structure [8,13–16]. Thus, collagen XVIII is essential for controlling blood vessel formation in the eye, and possibly an important component in the basement membrane zones of the entire vascular system [17]. The endothelial cells line the entire vascular system and are separated from the surrounding blood vessel layers by the basement membrane [18]. Following remodelling, damage and consequently degradation of the vascular basement membrane, the short isoform of collagen type XVIII may be affected and degraded, releasing measurable fragments of type XVIII collagen, as have been undertaken with other collagens [19,20].

The aim of this study was to quantify the basement membrane specifically for the endothelial cells, as this may have particular relevance to endothelial cell stability and rupture in haemophiliacs. For this, we developed a robust, specific and sensitive competitive ELISA assay, detecting the short isoform of collagen type XVIII (COL-18N), and assessed the clinical relevance of turnover of the basement membrane of the endothelial cells in patients diagnosed with HF arthropathy and corrected this to annual bleeding rate. This population of haemophilia patients has previously been characterized as having a relatively high annualized bleeding rate with active joint destruction [21] and a significant altered extracellular remodeling (Manon-Jensen et al., submitted), making it a well-suited population for quantifying remodelling of the vascular basement membrane.

## Materials and methods

### Patient samples

The patient cohort has previously been described [22]. In brief, serum was collected from 35 male HF patients aged 26 and over. This cut-off age was chosen as collagen turnover wears off at the closure of the growth plate at the age of app. 25 years [23]. The patients had a treatment history of either on-demand medication upon bleeding episodes or intake of a low dosage of prophylaxis of 5-10IU/kg recombinant FVIII, 2–3 times/week. Patients had varying degrees of HF arthropathy defined by the World Federation of Haemophilia Physical Examination Score (Gilbert Score) and by radiologic evaluation according to the Pettersson score. Both scores were assessed by trained personnel at the Department of Hematology, Peking Union Medical College Hospital, Beijing, China. Based on patient diaries the average ABR was 18.1 ranging from 2–46. Exclusion criteria were bleeding disorders other than haemophilia, human immunodeficiency virus infection, chronic obstructive pulmonary disease, medical history of joint disease or liver fibrosis, and treatment with anti-inflammatory biologics or steroids. Study participants were enrolled at the Department of Haematology, Peking Union Medical College Hospital, Beijing, China. The study was approved by the Peking Union Medical College Hospital, Chinese Academy of Medical Sciences, Ethics Review Board, with the serial number S-720. Signed informed consent was obtained from all subjects.

### Monoclonal antibody development for COL-18N

We used the first 10 amino acids of the N-terminal epitope of the short isoform of human type XVIII collagen α1 chain (excluding signal peptide, ^34’^EPERISEEVG^’43^) as peptide to generate monoclonal neo-epitope specific antibodies. Beijing Administration Office of Laboratory Animal and animal ethics committee of Nordic Bioscience approved the animal work. Generation of monoclonal antibodies was initiated by subcutaneous immunization of 6–8 week old Balb/C mice using 200μL emulsified Freund's complete adjuvant with 60μg peptide conjugated to keyhole limpet hemocyanin (KLH). Consecutive immunizations were done at 2-week intervals in Freund's incomplete adjuvant, until stable titer levels were reached. The mouse was boosted intravenously with 50μg immunogen in 100μL 0.9% sodium chloride solution and three days later the spleen cells were fused with SP2/0 myeloma cells (LGC Standards AB, Boras, Sweden) [24]. The hybridomas were grown in 96-well plates and monoclonal growth was ensured by limited dilution. Clones were screened against the specific epitope (EPERISEEVG), elongated peptide (AEPERISEEVG) and truncated peptide (PERISEEVG). The mAb producing clone, NB632-13H11/G5, was selected based on reactivity to above-mentioned peptides, and antibody purified using Protein G columns (GE Healthcare, Hilleroed, Denmark).

### COL-18N ELISA protocol

The competitive COL-18N ELISA was performed accordingly. A 96-well streptavidin-coated plate (Roche cat.: 11940279) was coated with 100μl/well 1.25ng/mL biotinylated synthetic peptide EPERISEEVG-K-Biotin dissolved in coating buffer (20mM Na_2_HPO_4_, 3.7mM KH_2_PO_4_, 137mM NaCl, 2.7mM KCL, 0.1% Tween20, 1% BSA, pH7.4) and incubated on a shaker for 30 min. at 20°C, 300 rpm. The plate was washed five times with washing buffer (20mM Tris, 50mM NaCl, pH 7.2). 20μL of the standard peptide (EPERISEEVG) or samples diluted in incubation buffer (20mM Na_2_HPO_4_, 3.7mM KH_2_PO_4_, 137mM NaCl, 2.7mM KCL, 0.1% Tween20, 1% BSA, 5% Liquid II (Osteocalcin EIA Puf-Liq, Roche Diagnostics) pH7.4) were added to appropriate wells, followed by 100μL/well monoclonal antibody NB632-13H11/G5, and incubated for 1hr at 20°C, 300 rpm. After washing, 100μl rabbit-anti-mouse antibody (Jackson, 315-035-045) was added 1:3000 dissolved in coating buffer and incubated 1hr at 20°C, 300rpm. After final five times wash, the wells were incubated with 100μL tetramethylbenzidine (TMB) (Kem-En-Tec cat. 438OH) at 20°C, 300rpm in the dark for 15 min., followed by the addition of 100μL/well-stopping solution (1% H_2_SO_4_). The colorimetric reaction was measured at 450nm with 650nm as reference, and a calibration curve was plotted using a 4-parametric mathematical fit model.

### COL-18N technical evaluation

Technical assay validation was performed according to international guidelines. The lower limit of detection (LLOD) was calculated as mean + 3x standard deviation (SD) determined from 21 zero samples (i.e., the assay buffer) (S1 Data). The upper limit of detection (ULOD) was determined as the mean - 3xSD of 10 measurements of undiluted standard peptide (1000ng/ml) (S1 Data). The lower limit of quantification (LLOQ) was determined by the lowest possible concentration with an imprecision of less than 30% (S1 Data). The intra- and inter-assay variations were calculated as the mean of the variation of seven human samples by 10 independent runs in duplicates (S2 Data). Dilution recovery was determined in a 2-fold dilution of two human serum and three human citrate plasma, calculated as percentage recovery of diluted matrices compared to undiluted ones (S3 Data). Spiking recovery was assessed in human serum and citrate plasma spiked with standard peptide at concentrations covering the entire measure range or by combining two samples of similar concentration in order to double the concentration (S4 Data). Spiking recovery was calculated as the measured amount percentage recovery of the theoretical amount. Interference by hemoglobin, lipemia, and biotin was determined by adding two-fold dilutions to a serum sample of known concentration (S5 Data). Concentrations started at 5 g/L hemoglobin, 5 g/L lipemia, and 40 ng/ml biotin (S5 Data). Recovery percentage was calculated with the normal serum sample as a reference. Analyte stability was determined for two healthy human serum samples and one healthy citrate plasma sample for four freeze-thaw cycles and calculated as the percentage recovery of the first freeze-thaw cycle (S6 Data). Same samples were tested at 2 hrs, 4 hrs and 24 hrs at 4°C and 20°C against non-stressed analytes (S7 Data). Finally, antibody specificity was assessed by a sanity check testing reactivity towards standard (EPERISEEVG), elongated (AEPERISEEVG), truncated (PERISEEVG) and de-selection peptides (EPQIDEKKK and CPERALERR) (S8 Data).

### Statistics

Correlations between serum COL18-N concentration and ABR were analyzed using Spearman rank correlation coefficient with GraphPad Prism v6 (GraphPad Software, La Jolla, CA, USA). Differences were considered statistically significant if p<0.05.

## Results and discussion

We developed and evaluated a novel competitive ELISA using a monoclonal antibody to detect COL-18N in human serum and plasma (citrate, EDTA, heparin) samples.

The main findings were:

- Serum COL-18N levels were correlated with ABR in HF patients.
- A technically stable assay for detecting COL-18N in human serum and human plasma with acceptable intra-inter assay variations and acceptable dilution and spike recoveries.

### Characterization of COL-18N ELISA

We developed a competitive COL-18N ELISA that can assess endothelial basement membrane degradation. The technical performance of the ELISA is summarized in Table 1, providing a measurement range from 4.8-671ng/ml, intra- and inter-variability at 7.4% and 13.1% respectively, dilution and spike recovery within 100±20% (Tables 2–4), and analytic stability (Tables 5 and 6) with no immunoassay interference. The normal concentration of COL-18N in serum (16.6ng/ml), plasma citrate (12.5ng/ml), EDTA plasma (13.2ng/ml), and heparin plasma (15.8ng/ml) was consistent regardless of matrices (S9 Data).

**Table 1:** Technical performance of COL-18N ELISA.

Measurements	Technical characteristics
LLOD	4.8 ng/ml
ULOD	671 ng/ml
LLOQ	7.3 ng/ml
Intra-assay variability	7.4% (accepted <10%)
Inter-assay variability	13.1% (accepted <15%)
Dilution recovery	within 100 ± 20%
Spiking recovery	within 100 ± 20%
Freeze-thaw stability (4 cycles)	within 100 ± 20%
Analyte stability (0–20 hrs at 4°C and 20°C)	within 100 ± 20%
Interference (Hgb, lipid, Biotin)	No interference
Human healthy serum (n = 10, mean value)	16.9 ng/ml
Human healthy plasma citrate (n = 10, mean value)	12.8 ng/ml
Human healthy plasma EDTA (n = 10, mean value)	13.8 ng/ml
Human healthy plasma heparin (n = 10, mean value)	15.6 ng/ml

**Table 2:** Dilution recovery of COL-18N assay.

COL-18N	HS1 (high)	HS2 (low)	HP1 (low)	HP2 (low)	HP3 (low)
**Undiluted**	100%	100%	100%	100%	100%
**Dilution 1:2**	99%	87%	99%	113%	82%
**Dilution 1:4**	103%	124%	-	-	98%
**Dilution 1:8**	120%	-	-	-	-
**Mean recovery**	107%	106%	99%	113%	90%

Percentage dilution recovery for the COL-18N assay using human serum (HS) and human plasma (HP). Values below LLOD are indicated by “—“. High level of COL-18N (high), low level of COL-18N (low).

**Table 3:** Spiking recovery.

COL-18N	Serum Recovery	Heparin plasma Recovery	Citrate plasma Recovery	EDTA plasma Recovery
**Matrix neat + spike**	92%	112%	108%	116%
**Matrix diluted 1:2 in buffer + spike**	106%	110%	103%	106%
**Matrix diluted 1:4 in buffer + spike**	107%	105%	95%	106%
**Matrix diluted 1:8 in buffer + spike**	102%	103%	110%	107%
**Mean recovery**	102%	108%	104%	109%

Spiking recovery was calculated as percentage recovery of measured spike (80 ng/ml standard peptide added) in buffer compared to measured spike in matrix, subtracted diluted matrix value.

**Table 4:** 1:1 spike recovery measured in two human serum samples.

COL-18N	Sample 1 (ng/ml)	Sample 2 (ng/ml)
**Serum neat ÷ spike**	34.9	34.3
**Serum neat + spike (35 ng/ml)**	75.9	79.9
**Recovery**	92%	87%

Spike recovery was calculated as the theoretical value of neat + spike compared to the measured value with neat + spike.

**Table 5:** Freeze-thaw stability.

Freeze/thaw cycle	Sample 1 (Human serum)	Sample 2 (Human serum)	Sample 3 (Human citrate plasma)	Recovery
1	100%	100%	100%	
2	116%	133%	114%	121%
3	104%	95%	117%	105%
4	116%	125%	99%	113%
			Mean recovery	113%

Analyte stability was assessed in two human serum and one plasma sample in four freeze/thaw cycles. All data are shown as mean percentage recovery compared to 1 freeze/thaw cycle.

**Table 6:** Analyte stability measured at different time points and temperatures.

Time	Temperature	Sample 1 (Human serum)	Sample 2 (Human serum)	sample 3 (Human plasma)	Mean recovery
**0 hrs**	**-**	100%	100%	100%	-
**2 hrs**	**4°C**	101%	104%	94%	100%
	**20°C**	87%	109%	103%	99%
**4 hrs**	**4°C**	94%	85%	106%	95%
	**20°C**	105%	111%	95%	104%
**24 hrs**	**4°C**	111%	119%	102%	111%
	**20°C**	90%	101%	115%	102%

Two human serum and one plasma sample were measured after 2 hrs, 4 hrs and 4 hours at 4°C and 20°C. All data are shown as mean percentage recovery compared to non-stressed analytes (0 hrs).

**Fig 2:**
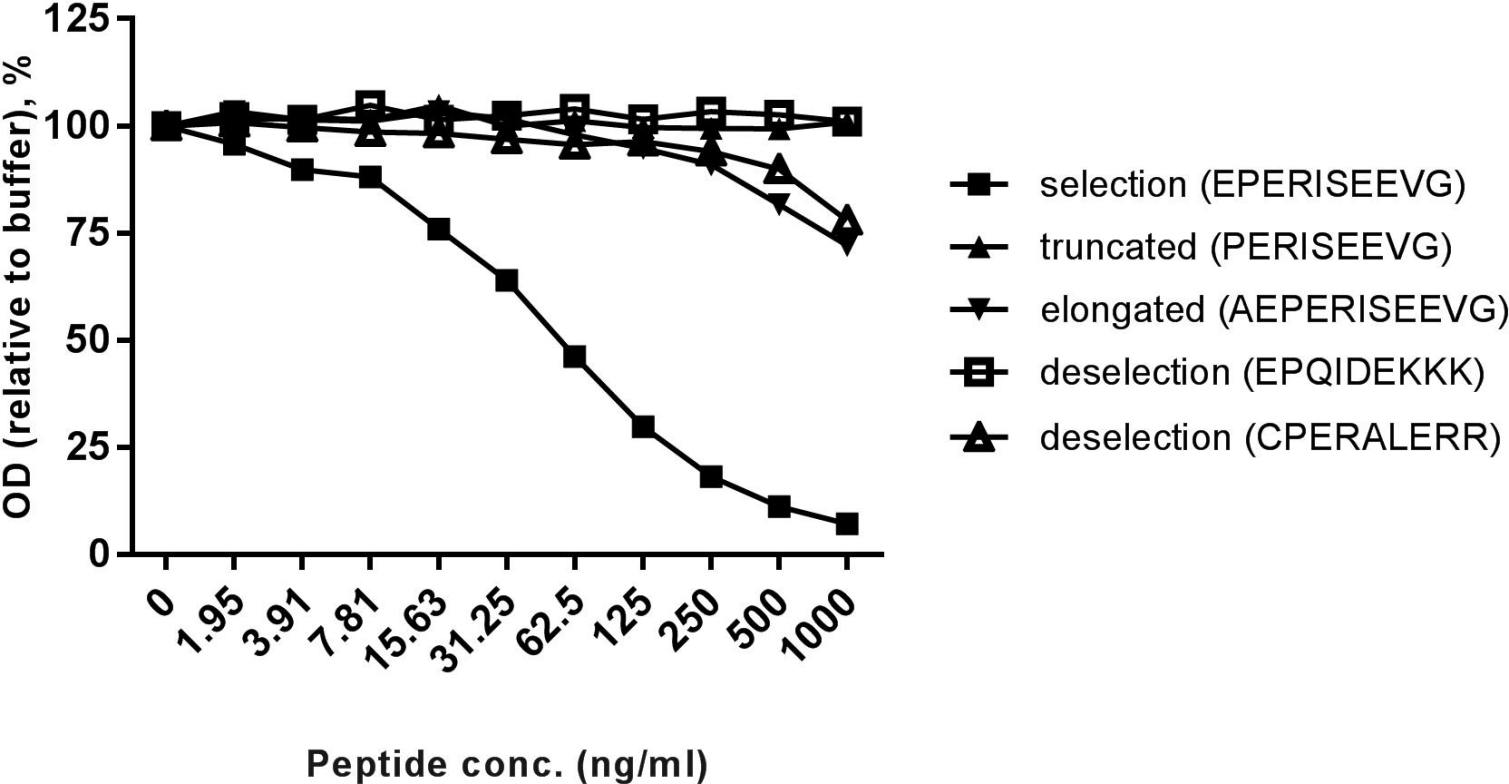
COL-18N antibody specific reactivity against type XVIII collagen. Monoclonal NB632-13H11/G5 antibody reaction towards standard peptide, truncated, elongated and de-selection peptides. The antibody has high reactivity towards standard peptide and no or minimal cross-reactivity with the other peptides.

The NB632-13H11/G5 antibody specially recognized the first 10 amino acids of N-terminus type XVIII collagen α1 chain, short isoform (selection) (Fig 2). The antibody showed no or minimal reactivity towards related peptides, indicating a high specificity (Fig 2). Possible cross-reactivity with N-terminus of intermediary and long forms of collagen type XVIII is not plausible, since the three isoforms of collagen type XVIII are encoded by the *COL18A1* gene by the use of two different promoters and alternative splicing (Fig 1B). As a result N-terminus of the short isoform becomes entirely different from N-terminus of the other two isoforms. The technical evaluation of the competitive COL-18N ELISA revealed a stable sensitive assay with high specificity towards the N-terminus of vascular form of type XVIII collagen including high accuracy and precision of the assay.

### COL-18N correlates to annual bleeding rate in HF patients

The haemorrhagic disorder haemophilia manifests clinically by repeated haemarthrosis resulting in unavoidable arthropathy in the absence of adequate treatment. One cardinal feature of medicinal intervention in haemophilia is lowering of ABR, albeit objective quantifiable parameters with high resolution are lacking.

The bleeding severity of haemophilia is generally inversely proportional to the degree of FVIII/IX activity in the plasma, although substantial variability in bleeding tendencies is well-known. Reduced spontaneous bleeding and lower requirements of factor concentrates are reported in a subset of 10–15% of severe HF patients [25,26]. Also, development of inhibitors in non-severe HF patients may heighten the bleeding phenotype considerably [27].

Bleeding phenotype may be further compromised by large discrepancies amongst the FVIII assays caused by standardization of the assays [28] and may even be influenced by the type of FVIII concentrates used during therapy [29,30]. Other assays, like thrombin generation, correlates to the bleeding phenotype in HF patients [31], but is inconsistent in HF patients with FVIII inhibitors despite the occurrence of thrombin generation [32].

In haemophilia, consequent to *i*) endothelial cell damage *ii*) bleeding and *iii*) delayed clotting and wound healing, the endothelial remodelling contributing to clinical symptoms of haemophilia and pathophysiological disease representation may be affected. After endothelial cell damage, blood and entry of inflammatory cells may cause extensive vascular extracellular matrix and basement membrane remodelling, where collagen is degraded and new collagen synthesized.

In a F8^-/-^ rat model of haemophilia and induced haemarthrosis, we have previously shown that F8^-/-^ rats had significantly higher concentrations of basement membrane biomarkers of type IV collagen degradation and formation compared to wild-type littermate controls, and that biomarkers of collagen turnover of both the basement membrane and interstitial matrix were significantly altered following induction of a knee bleed [33]. This suggests that endothelial rupture and hemorrhage with delayed clotting results in altered turnover of the extracellular matrix.

**Fig 3:**
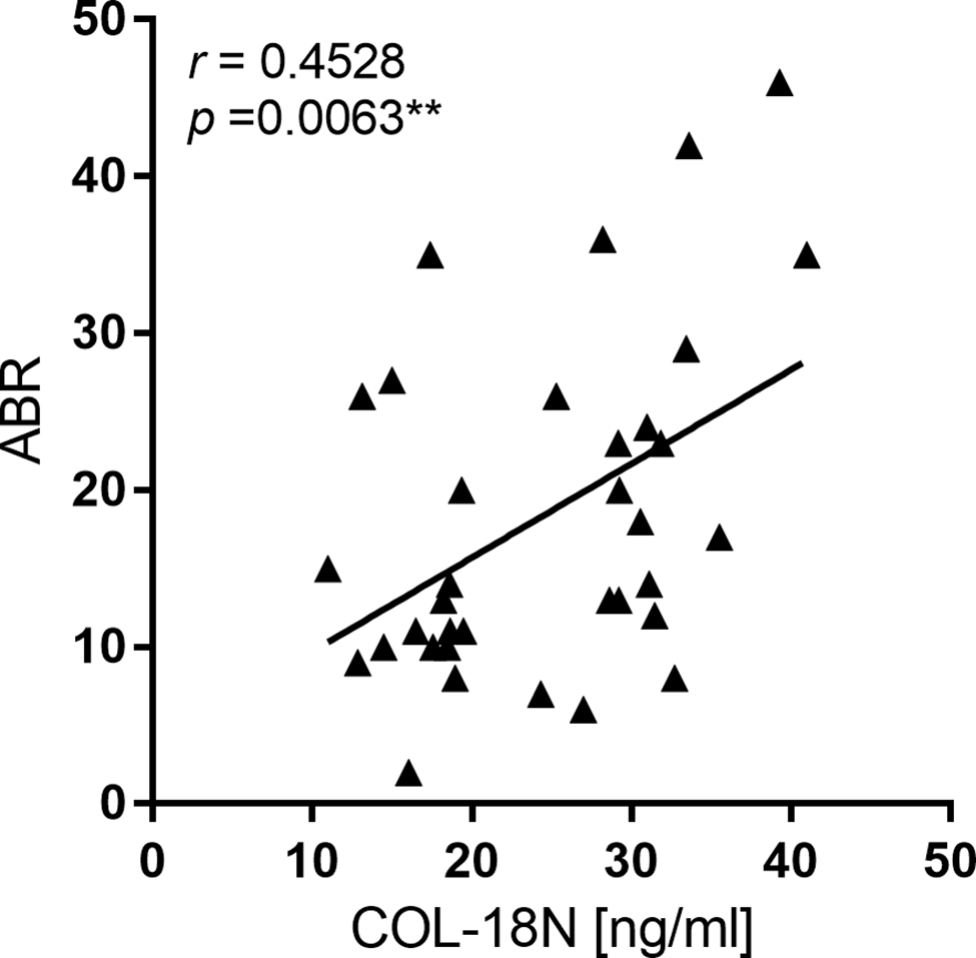
Biomarker COL-18N correlate with ABR. Serum from 35 male HF patients aged 26 and over was measured with the COL-18N ELISA. Correlations between vascular endothelial type XVIII collagen concentration and ABR were analyzed using Spearman rank correlation coefficient and shown r = 0.45, p<0.006. Differences between ABR and COL-18N levels were considered statistically significant if p<0.05 and significant levels are displayed as: * = p<0.05; ** = p<0.01, and *** = p<0.001.

Herein, we found vascular endothelial type XVIII collagen to correlate with ABR in HF patients (Fig 3, r = 0.45, p<0.006, S10 Data,). Basement membrane turnover in haemophilia patients have been reported upregulated relative to the control group as indicated by a biomarker of type IV collagen degradation [34]. Objective biomarkers of pathological processes, like those of degraded type XVIII collagen that associates with ABR, may assist in benchmarking treatments, monitor patients and consequently assist in drug development for the benefit of patients and payers.

We acknowledge that there is limitations to this study. The actual ABR is extremely hard to quantify, consequently we do not know whether the correlation of 0.45 would have been higher if the gold standard was more accurate.

## Conclusion

In summary, the data combined suggests that the technically robust COL-18N biomarker can be related to pathologies involving vascular basement membrane degradation and remodelling, which affects degradation of the short isoform of type XVIII collagen. In addition, the data enables the COL-18N biomarker to evaluate ABR for optimal treatment and monitoring of patients to prevent the development of arthropathy.

## Supporting information

S1 Data LLOD, ULOD, ULOQ. (XLSX)

S2 Data Intra- and inter-assay variations. (XLSX)

S3 Data Dilution recovery. (XLSX)

S4 Data Spiking recovery. (XLSX)

S5 Data Interference. (XLSX)

S6 Data Freeze-thaw cycles. (XLSX)

S7 Data Analyte stability. (XLSX)

S8 Data Antibody specificity assessed by reactivity towards standard, elongated, truncated and de-selection peptides. (XLSX)

S9 Data Serum levels of type XVIII collagen (COL-18N) in serum, plasma EDTA, plasma citrate and plasma heparin samples of healthy donors. (XLSX)

S10 Data Serum levels of type XVIII collagen (COL-18N) and ABR for patients with haemophilia. (XLSX)
